# Demographic patterns of cutaneous T-cell lymphoma incidence in Texas based on two different cancer registries

**DOI:** 10.1002/cam4.472

**Published:** 2015-07-01

**Authors:** Ivan V Litvinov, Michael T Tetzlaff, Elham Rahme, Michelle A Jennings, David R Risser, Pamela Gangar, Elena Netchiporouk, Linda Moreau, Victor G Prieto, Denis Sasseville, Madeleine Duvic

**Affiliations:** 1 Division of Dermatology, McGill UniversityMontreal, Quebec, Canada; 2 Section of Dermatopathology, Department of Pathology, The University of Texas MD Anderson Cancer CenterHouston, Texas; 3 Division of Clinical Epidemiology, McGill UniversityMontreal, Quebec, Canada; 4 Cancer Epidemiology and Surveillance Branch, Texas Cancer Registry, Department of State Health ServicesAustin, Texas; 5 Department of Dermatology, The University of Texas MD Anderson Cancer CenterHouston, Texas

**Keywords:** Cutaneous T-cell lymphoma (CTCL), demographic patterns, geographic clustering, mycosis fungoides, Sézary syndrome

## Abstract

Cutaneous T-cell lymohomas (CTCLs) are rare, but potentially devastating malignancies, with Mycosis fungoides and Sézary Syndrome being the most common. In our previous study, we identified and described regions of geographic clustering of CTCL cases in Texas by analyzing ∼1990 patients using two distinct cancer registries. In the current work, we describe in detail demographic patterns for this malignancy in our study population and apply logistic regression models to analyze the incidence of CTCL by sex, race, age, and clinical stage at the time of diagnosis. Furthermore, using Fisher's exact test, we analyze changes in incidence over time in the identified Houston communities with unusually high CTCL incidence. While CTCL primarily affects Caucasian individuals >55 years old, we confirm that it presents at a younger age and with more advanced disease stages in African-American and Hispanic individuals. Also, we demonstrate a significant increase in CTCL incidence over time in the identified communities. Spring, Katy, and Houston Memorial areas had high baseline rates. Furthermore, a statistically significant disease surge was observed in these areas after ∼2005. This report supplements our initial study documenting the existence of geographic clustering of CTCL cases in Texas and in greater detail describes demographic trends for our patient population. The observed surge in CTCL incidence in the three identified communities further argues that this malignancy may be triggered by one or more external etiologic agents.

## Introduction

Cutaneous T-cell lymphomas (CTCLs) are rare, but potentially devastating malignancies that have distinct clinical and histological features. Mycosis fungoides (MF), primary cutaneous anaplastic large cell lymphoma and a leukemic form, Sézary Syndrome (SS), are the most common variants and account for ∼80% of all cutaneous lymphomas 1. Epidemiologic studies based on the Surveillance, Epidemiology, and End Results (SEER) databases established that until recently CTCLs were on the rise in the United States 2 and multiple reports have shown a ∼threefold increase in CTCL incidence during the last 25–30 years 3,4. Based on recent analyses, the incidence of this cancer during 2000–2010 years reached ∼10 cases per million individuals per year in the United States 2. Similarly, increase in CTCL incidence has been observed in other parts of the world 5–7. This malignancy often presents at an older age (i.e., >55 years old) in Caucasians, but in African-American, Hispanic, and Middle-Eastern individuals studies have shown an earlier age at presentation with a more aggressive clinical course 4,5,8.

The molecular pathogenesis of CTCL is only partially understood. MF is hypothesized to arise from antigen-stimulated helper/memory T cells, but the antigen remains unknown and would likely differ among patients. Many studies have attempted to elucidate the genetic basis of this cancer 9–14, whereas others strived to identify cancer-initiating cells for MF and SS 15. Previous studies have demonstrated that CTCL can occur in married couples 16–18 and may cluster in families 19,20. Recent investigations demonstrated geographic clustering of CTCL cases in the Västernorrland county of Sweden 21 and Pittsburgh, Pennsylvania 22 therefore implying possible existence of an environmental trigger for this cancer. Prior studies have looked for a viral, chemical, or an occupational disease trigger, but failed to yield any conclusive etiologic agents 23–25. Some cases of MF have been associated with hydrochlorothiazide and other medications 26. Some patients with smoldering HTLV-1 associated adult T-cell lymphoma present with MF-like skin lesions 27,28, but based on other studies, retroviruses have not been identified in the vast majority of MF cases 29,30.

In our recent study, based on two distinct cancer registries, we have, for the first time, documented geographic clustering of patients in several communities across Texas 31. This included the cities of Beaumont, Katy, Spring, and Memorial area of Houston, where CTCL incidence rates were 5–20 times higher than the expected population rate. Furthermore, our analysis demonstrated that two densely populated adjacent zip codes (79936 and 79928) near El Paso, Texas were completely spared by CTCL during 1995–2010 31. El Paso is located in a hot desert climate adjacent to the borders of New Mexico and Mexico. Hispanic individuals represent >80% of population in this area. Notably, El Paso, TX was documented to be one of the sunniest cities in the country 32. El Paso is the fourth sunniest city in the United States and first in Texas with 84% annual sunshine, which could be a protective or a therapeutic factor for MF 32.

In this work using the MD Anderson Cancer Center (MDACC) CTCL clinic patient database together with the statewide population-based Texas Cancer Registry (TCR), we expand on our initial observations and describe the patterns of demographic incidence for CTCL in our patient population. We also describe the changes in incidence rates over time in identified geographic clusters.

## Materials and Methods

### Patient demographics and chart review

This study was approved by the MDACC IRB (IRB protocols: PA12-0497, PA12-0267 and Lab97-256). All patients signed an IRB-approved consent 33. Patients in the MDACC CTCL clinic database were prospectively invited to participate in a research study on the pathogenesis of CTCL with a participation rate of >90%. Sex, race, date of diagnosis as well as age, clinical stage, and residential address at the time of diagnosis were analyzed for patients seen in the clinic between the years 2000 and 2012 (i.e., MDACC database).

The TCR is a population-based registry that collects data on all cancers, including CTCL, for the entire state of Texas. Hence, to confirm our results we obtained de-identified data from this public database. ICD-O codes 9700/3, 9709/3, 9701/3 and ICD-10 codes C84.0, C84.1, C84.8 were used to identify cases of CTCL diagnosed statewide from 1996 to 2010 (dates of data availability). Data were provided for the entire state and for each individual zip code. The TCR was not able to provide data by clinical stage at the time of diagnosis. In our previous study, we documented that MDACC has a catch rate of ∼52% for the entire state of Texas when compared with the TCR database, whereas for overlapping zipcodes, 87% correlation rate was observed between the two databases 31.

### Statistical analyses

Unless otherwise specified, analysis of the complete data on all patients seen at the MDACC CTCL clinic from 2000 to 2012 is presented throughout the study. Incidence rates and 95% confidence intervals (CI) were calculated and reported. Unless otherwise specified, 2000 and 2010 US Census data were used for all population analyses, where results before 2005 were compared to the year 2000 US Census, whereas results after 2006 were compared to the 2010 US census. Confidence intervals were based on Poisson distributions. Graphs were used to visually report these rates. Incidence rates were compared to the expected rates using Chi Square or Fisher's exact tests as appropriate. Demographic characteristics including race (Caucasian, African-American, Hispanic, and other) and age groups (<40, 40–59, and ≥60 years old) were assessed and reported. To examine whether differences exist between men and women or different races, logistic regression models were conducted accounting for age, sex, race, and stage of the disease at the time of diagnosis. Standard model selection procedures were used to select the final models 34.

## Results

The MDACC multidisciplinary CTCL clinic has been in existence since 1987 and has a high rate of referral from Texas and the surrounding states. During the study period (2000–2012), 1047 new CTCL patients were seen in our clinic with an average of ∼80 new cases evaluated and treated each year from Texas. In most cases, patients received their initial diagnosis elsewhere and were referred to our clinic for further evaluation and management. Each patient's slides were reviewed by an MDACC dermatopathologist to confirm their diagnosis.

The TCR collects data on all cancers, including CTCL, for the entire state of Texas. On the basis of data availability, we were able to obtain de-identified data from 1996 through 2010. During this time 1990 new cases of CTCL were recorded in the registry with ∼132 cases on average being documented each year. The overall annual incidence rate for the state of Texas for 1996 through 2010 was 5.77; 95% CI [5.52, 6.03] per million individuals per year.

### Analysis of patient demographic characteristics

**Figure 1:**
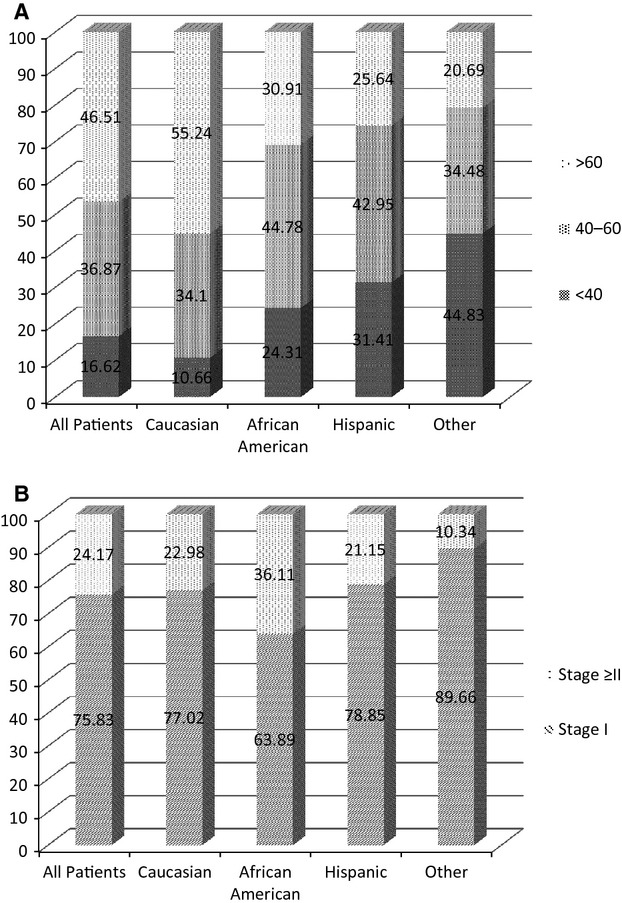
Graphic representation of patient characteristics by race (MDACC database results). (A) Patient age of diagnosis distribution by race. (B) Clinical disease stage at the time of diagnosis by race.

We examined the demographic characteristics of our patient population based on the MDACC database. Initial analysis of patient characteristics revealed that they came from various racial groups that were reflective of the demographic representation of the state (Table1). The mean age of diagnosis for all patients was 56.3 ± 16.6 years. Analysis by race demonstrated that the mean age of diagnosis was 60.0 ± 14.8 years for Caucasian, 49.3 ± 16.9 years for African-American and 47.4 ± 17.8 years for Hispanic patients. The observed age differences between ethnic groups support the findings of previous reports indicating that African-American and Hispanic patients have an earlier onset of the disease 4,8, and are further supported by a logistic regression analysis (Table2). Graphic analysis of age at the time of diagnosis by ethnicity is presented in Figure1A. The above findings are also supported by the analysis of the TCR database, which showed almost identical patient distribution according to gender and race (Table3). The overall average age of diagnosis according to the TCR database was 58.7 ± 17.2. Similar to the MDACC findings, the age of diagnosis for Caucasians was 62.0 ± 15.5, whereas for African-Americans, Hispanic, and Pacific-Islander nationalities the mean ages were 52.2 ± 18.6, 52.8 ± 17.9, and 45.9 ± 17.0, respectively. The observed age differences between ethnic groups, based on the TCR database, were also compared using logistic regression analysis (Table4).

**Table 1:** Clinical characteristics of CTCL patients in the study based on MDACC databases. Tables adopted from 31

MDACC database	State of Texas	
	*n*	%
Number of patients	1047	
Age at diagnosis	56.3 ± 16.6	
Sex		
Female	491	46.9
Male	556	53.1
Race		
Caucasian	718	68.6
African-American	144	13.8
Hispanic	156	14.9
Other	29	2.8
CTCL clinical stage		
I	794	75.8
II	112	10.7
III	32	3.1
IV	109	10.4

**Table 2:** Logistic regression analysis of patient characteristics associated with African-American, Hispanic, and other races versus Caucasian ethnicity using MDACC databases

Logistic regression analysis odds ratio [95% confidence interval]			
Patient characteristics	African-American versus caucasian	Hispanic versus caucasian	Other ethnicities versus caucasian
Age			
<40	5.71 [3.18, 10.25]	7.17 [4.21, 12.22]	12.04 [3.98, 36.41]
40–59	2.69 [1.68, 4.30]	2.74 [1.74, 4.31]	3.07 [1.03, 9.16]
≥60	1 (reference)	1 (reference)	1 (reference)
Clinical disease stage			
>II	2.83 [1.81, 4.42]	1.25 [0.78, 2.01]	0.64 [0.18, 2.20]
I	1 (reference)	1 (reference)	1 (reference)
Sex			
Male	0.43 [0.29, 0.66]	0.76 [0.52, 1.11]	1.18 [0.53, 2.66]
Female	1 (reference)	1 (reference)	1 (reference)

**Table 3:** Clinical characteristics of CTCL patients in the study based on TCR databases. Tables adopted from 31

TCR database	State of Texas	
	*n*	%
Number of patients	1990	
Age at diagnosis	58.7 ± 17.2	
Sex		
Female	892	44.8
Male	1098	55.2
Race		
Caucasian	1308	65.7
African-American	318	16.0
Hispanic	303	15.2
Asian pacific	24	1.2
Other	37	1.9

**Table 4:** Logistic regression analysis of patient characteristics associated with African-American, Hispanic, and other races versus Caucasian ethnicity using TCR databases

Logistic regression analysis odds ratio [95% confidence interval]			
Patient characteristics	African-American versus caucasian	Hispanic versus caucasian	Asian-pacific versus caucasian
Age			
<40	3.99 [2.81, 5.65]	4.30 [3.01, 6.14]	12.15 [4.38, 33.70]
40–59	1.79 [1.36, 2.37]	1.97 [1.49, 2.62]	2.24 [0.75, 6.7]
≥60	1 (reference)	1 (reference)	1 (reference)
Sex			
Male	0.71 [0.55, 0.91]	0.91 [0.70, 1.18]	0.76 [0.33, 1.72]
Female	1 (reference)	1 (reference)	1 (reference)

Furthermore, consistent with previous reports 35, the MDACC database demonstrated that more African-American patients (36.1%) presented with advanced disease stages (i.e., stage ≥ II) compared to Caucasians (23.0%) or Hispanics (21.2%). These findings were further confirmed by a logistic regression analysis odds ratio (OR) of 2.83 [1.81, 4.42] (Tables2 and 5). Graphical analysis of the clinical disease stage at the time of diagnosis by race is presented in Figure1B. Also, males were 61% more likely than females to present with advanced disease OR 1.61 [1.17, 2.22] (Tables5 and 6).

**Table 5:** Patient characteristics associated with clinical disease stage ≥ II versus stage = I at the time of diagnosis based on the MDACC database results (2000–2011)

Logistic regression analysis	
Patient characteristics	Odds ratio [95% confidence interval]
Age	
<40	0.30 [0.17, 0.53]
40–59	0.57 [0.40, 0.80]
≥60	1 (reference)
Sex	
Male	1.61 [1.17, 2.22]
Female	1 (reference)
Race	
African-American	2.83 [1.81, 4.42]
Hispanic	1.25 [0.78, 2.01]
Other	0.64 [0.19, 2.22]
Caucasian	1 (reference)

**Table 6:** Patient characteristics associated with male versus female sex based on the MDACC database results (2000–2011)

Logistic regression analysis	
Patient characteristics	Odds ratio [95% confidence interval]
Age	
<40	0.70 [0.46, 1.05]
40–56	0.81 [0.60, 1.09]
≥60	1 (reference)
Clinical disease stage	
≥II	1.61 [1.17, 2.22]
I	1 (reference)
Race	
African-American	0.43 [0.29, 0.66]
Hispanic	0.76 [0.52, 1.11]
Other	1.18 [0.53, 2.66]
Caucasian	1 (reference)

### Change in rate of incidence over time

**Figure 2:**
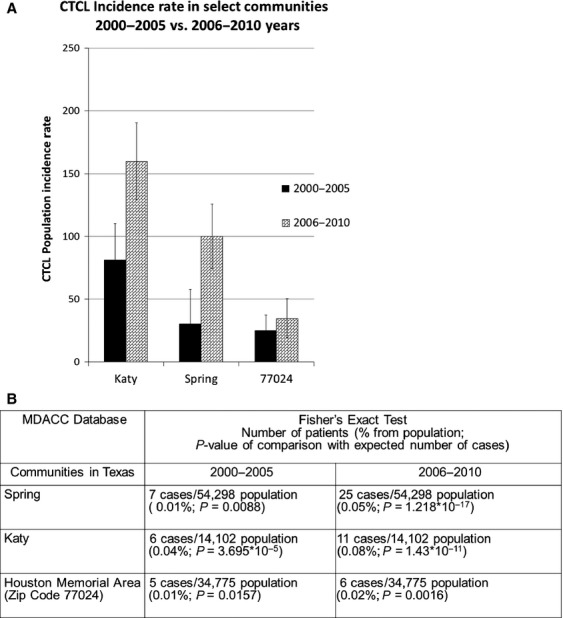
Incidence rates of CTCL in the identified communities over time. (A) Graphical comparison of CTCL incidence rate for communities with high CTCL incidence during 2000–2005 versus 2006–2010 years (MDACC database results). (B) Fisher's exact test comparison of CTCL incidence in Houston communities to population rate of 6.4 cases per million per year.

In our previous report, we documented three areas of geographic clustering of CTCL in Houston: Katy, Spring, and Memorial area. Based on the 2000–2010 MDACC database results, the overall incidence rates were 136 cases per million per year in Katy, 52 cases per million per year in Spring and 34 cases per million per year in Houston Memorial area (zip code 77024). In comparison, the overall incidence rate for Texas and Houston were 5.8 and 6.4 cases per million per year, respectively. Analysis of the incidence rate over time documented that while these communities had a high baseline rate of disease, there was a dramatic increase in the incidence of CTCL after ∼2005 (Fig.2A). The comparison of incidence rate before and after 2005 documented a significant jump in incidence rate. This was further supported by Fisher's exact test comparing the incidence of CTCL in these communities to the documented incidence rate in Houston (Fig.2B). Calculated *P* values signifying deviation from the norm were highly statistically significant and are presented in Figure2B for each community.

## Discussion

The current report supplements our initial study documenting the existence of geographic clustering of CTCL cases in Texas and describes in greater detail demographic trends for our patient population. This study further highlights that while in Caucasian patients CTCL principally affects individuals ≥60 years old, in African-American and Hispanic individuals it presents at a much younger age (i.e., during 40s). Furthermore, in African-Americans and in men CTCL is often diagnosed at an advanced clinical stage (≥stage II). Also, we demonstrate a significant increase in CTCL incidence over time in the identified communities. While Spring, Katy, and Houston Memorial areas had a high baseline rate, a significant disease surge was observed after 2005. This finding lends further support to the notion that this malignancy may be triggered by an external etiologic agent.

Previous epidemiologic studies based on the Surveillance, Epidemiology and End Results (SEER) databases established that CTCL is on the rise in the United States and around the world 36. Similarly, a recent increase in these cancers has been identified in Saudi Arabia and Kuwait, where MF often affects children and adolescents 37,38. Our findings suggest that geographic mapping of patients by various CTCL centers will reveal additional areas of geographic clustering. Further analysis of these CTCL “endemic” areas will hopefully identify external causes and may help us prevent this cancer in the future.

Notably, parallels can be drawn with other rare malignancies, where increased incidence in certain populations and/or geographic areas made it possible to identify a definitive trigger. For example, studies assessing the prevalence of mesotheliomas in the mining regions of South Africa and Quebec, Canada, established asbestos as a critical factor responsible for this deadly disease 39,40. Similarly, a study of a small arsenic mining town in Prussia in 1898, where chronic poisoning of drinking water supply occurred, helped establish the link between arsenic and the occurrence of arsenical keratoses and skin squamous cell carcinomas 41. Thus, epidemiologic studies showing regional clustering of patients may help identify environmental triggers for CTCL and other rare cancers leading to earlier detection or even prevention.
